# The effects of posterior cruciate ligament rupture on the biomechanical and histological characteristics of the medial collateral ligament: an animal study

**DOI:** 10.1186/s13018-021-02443-0

**Published:** 2021-05-21

**Authors:** Wen-qing Xie, Miao He, Yu-qiong He, Deng-jie Yu, Hong-fu Jin, Fang Yu, Yu-sheng Li

**Affiliations:** 1 Department of Orthopaedics, Xiangya Hospital, Central South University, 87 Xiangya Road, Changsha, 410008 Hunan China; 2 National Clinical Research Center for Geriatric Disorders, Xiangya Hospital, Central South University, 87 Xiangya Road, Changsha, 410008 Hunan China

**Keywords:** PCL rupture, MCL, Collagen fiber, Elastic modulus, Micro-hardness

## Abstract

**Background:**

To investigate the effect of complete rupture of the posterior cruciate ligament (PCL) on the biomechanics and histology of the medial collateral ligament (MCL).

**Materials and methods:**

Seventy-two male rabbits were randomly divided into two groups: the ruptured group was treated with complete PCL amputation, while the intact group was only subjected to PCL exposure without amputation. Eighteen rabbits were randomly sacrificed at 8, 16, 24, and 40 weeks after the operation, and their specimens were processed for mechanical tensile testing, nano-indentation experiments, hematoxylin-eosin (HE) staining, and picrosirius-polarization staining.

**Results:**

There was no significant difference in the length and maximum displacement of the MCL between the ruptured group and the intact group at each time point. The maximum load of the ruptured group was significantly smaller than that of the intact group at 40 W. The elastic modulus and micro-hardness of the ruptured group increased significantly at 24 W and decreased significantly at 40 W. At 16 W and 24 W after PCL rupture, the number of type I collagen fibers and type III collagen fibers in the MCL of the ruptured group was significantly increased compared with that of the intact group. While the type I collagen fibers of the ruptured group were significantly decreased compared with the intact group at 40 W, there was no significant difference in type III collagen fibers between the ruptured group and the intact group.

**Conclusion:**

PCL rupture has no significant effect on the mechanical and histological properties of MCL in a short period of time under physiological loading, but the histological and mechanical properties of MCL decrease with time.

## Introduction

The PCL is an indispensable ligament for maintaining the stability of the knee joint. This ligament is the most powerful in the knee joint and is connected to the femur and tibia in the form of a hinge [1, 2]. The PCL corresponds to the anterior cruciate ligament (ACL), which mainly prevents the tibia from moving backward relative to the femur. It has been reported that the average age of PCL injuries is 27.5 9.9 years, of which traffic accidents (45%) and sports injuries (40%) are the most common causes of injury [3]. However, the latest epidemiological results show that physical activity (38.8%) is the main factor of PCL injury, followed by traffic injury (35%) [4]. Any force that causes PCL stress can cause PCL injury, and its injury mechanism [5, 6] is usually the excessive application of force from front to back to the tibia, which often occurs in the flexion position.

According to the degree of relaxation, PCL injury can be divided into 3. At present, most scholars have reached a consensus on degree III injuries, that is, conservative treatment [7,9]. However, the treatment of degree III injury is still controversial. Some scholars believe that timely surgery can delay the occurrence of osteoarthritis (OA). Some people think that surgical reconstruction may not be able to prevent the occurrence of OA. However, it is still considered that surgical reconstruction should be performed when the PCL is in grade III injury [10, 11]. Unfortunately, most of the current treatment options for PCL injury are controversial. There is no effective and rigorous randomized controlled trial to compare various treatment methods or complete and rigorous guidelines for PCL injury to guide clinicians.

Because PCL rupture is mostly caused by strong violence, it often combines with other structures inside and outside the joint. Schulz et al. [3] found that only 47% of patients had simple PCL injuries. Petrigliano et al. [12] reported that 96.5% of PCL injuries were associated with other ligament injuries, including anterior cruciate ligament, lateral collateral ligament, or lateral stable structure injury. The biomechanical or histological effects of PCL rupture on the medial femoral condyle [13], the lateral femoral condyle [14], the medical tibial plateau [15], and the medical meniscus [16] have been studied by scholars. However, the effect of PCL rupture on the MCL is still lacking, and most of the existing studies focus on the ACL.

Therefore, in this study, mechanical tensile testing and nano-indentation technology were used to study the MCL after PCL rupture. At the same time, the change in the mechanical properties of the ligament and the relationship between them were discussed at the micro- and macro-levels. In addition, the histomorphology of MCL, the quantity of type I and type III collagen, and the ratio of type I and type III collagen were observed by histological methods.

## Materials and methods

### Materials

#### Experimental animals

Seventy-two 2-month-old male rabbits with an average body weight of 2.5 0.4 kg were provided by the experimental animal Department of Xiangya Hospital, Central South University. The animal experiments were carried out in accordance with relevant guidelines and regulations and were approved by the medical ethics committee of Xiangya Hospital of Central South University (No: 201908799). The adaptation before intervention was as follows: feeding environment for 7 days; ambient temperature (21 3) C; relative humidity (55 5)%, light-dark cycle of 24 h and normal circadian rhythm. The diet and diarrhea of the animals were observed every day.

#### Main experimental equipment 

An MTS 858 system (provided by the key Mechanics Laboratory of the Ministry of Education, School of Materials Science and Engineering, Central South University, manufactured by MTS company, USA), an ultra-nano-hardness tester (unht), and an OPX system (provided by the National Key Laboratory of powder metallurgy of Central South University and manufactured by CSM instrument company of Switzerland) were used.

#### Main reagents

Pentobarbital sodium (subpackaged by the Shanghai Chemical Reagent Company of China Pharmaceutical Group) was prepared into a 3% (0.03 mg/kg) solution for standby, and penicillin powder injection for injection (North China Pharmaceutical Co., Ltd.) and 0.1% sirius red picric acid staining solution (Beijing Hyde Biological Preparation Co., Ltd.) were also purchased.

### Methods

#### Experimental group

Seventy-two rabbits were randomly divided into an experimental group and a control group. The observation time points were 8 W, 16 W, 24 W, and 40 W after modeling. Eighteen rabbits were randomly sacrificed at each time point for biomechanics and histological study. Six of them were subjected to mechanical tensile testing, six were subjected to nano-indentation testing, and the other six were subjected to histological analysis. A total of 48 rabbits were studied for biomechanics, and 24 rabbits were studied for histology.

#### Model preparation

The method used was the same as that in our previous study [15, 16]. In short, rabbits were injected with 3% pentobarbital solution (0.03 mg/kg) through the ear vein. After anaesthesia was achieved, the rabbits were fixed in the supine position. The stability of the knee joint was checked by anterior and posterior drawer and medial and lateral turnover stress tests. Skin preparation, disinfection, and towel spreading were carried out in the bilateral knee joint area. In the experimental group, the skin, subcutaneous tissue, and medial patellar retinaculum were incised layer by layer. The patella was dislocated laterally, and the PCL was exposed and completely removed. The intra-articular tissues were protected during the operation, and hemostasis was carefully performed. After washing with normal saline and suturing layer by layer, a gauze bandage was used to bandage without fixation. The operation procedure of the control group was the same as that of the experimental group, but the PCL was only exposed without being removed. After the operation, the rabbits were fed in cages and had free drinking water. The diet, diarrhea, and wound infection of the rabbits were observed every day. Penicillin 80 104 U was intramuscularly injected once a day for 3 days. The rabbits were killed by air embolism at each time point, and the specimens were collected.

#### Biomechanical observation indexes

The lateral collateral ligament, anterior PCL, and meniscus of the rabbit knee joint were removed, only the femur MCL tibia complex (FMTC) was reserved, and approximately 6 cm of the tibia and femur was reserved. Observations: (1) the length of the MCL was as follows: the initial length of the MCL of 48 experimental rabbits was measured with a Vernier caliper (the measurement accuracy was 0.01 mm), and the average value was obtained by measuring 3 times; (2) the mechanical tensile test was performed as follows: the method used in the previous study was the same [17]. In short, the two ends of the FMTC of 24 rabbits were fixed with a homemade tensile test fixture and then placed on a dynamic tension torsion combined testing machine to make the longitudinal axis of the ligament consistent with the direction of the tensile force line. During the stretching process, a static preload of 0.5 N was applied for 5 minutes, and then, the maximum load was loaded and unloaded at a rate of 5 mm/min at 0.5% of the maximum load 20 times. Then, the maximum load tensile test was conducted at 5 mm/min. The maximum loading load and displacement of the MCL were recorded. (3) The nano-indentation experiment was conducted as follows: the method used was the same as that described in a previous study [18]. In short, the MCL specimens of 24 experimental rabbits were cut parallel to the longitudinal axis with a frozen microtome, placed on a homemade slide, and fixed on the stage of the ultra-nano-indentation instrument. The smooth surface area was manually searched under an optical microscope at 4000 times magnification, the depth was measured with a diamond probe, and the indentation images were collected. Under constant strain rate loading mode, the maximum load was 500 N, the loading and unloading speed was 50 N/s, and the holding and unloading times were both 10 s. Five points in the middle area of the sample were selected for the indentation test, and the distance between the points was more than 20 times the indentation diameter. The load depth curve was recorded, and the micro-hardness and elastic modulus were obtained from the unloading stage.

#### Histological observation index

The MCL specimens of 24 rabbits were observed as follows: (1) HE staining: the MCL specimens were washed with normal saline, fixed in 4% paraformaldehyde solution for 24 h, decalcified, dehydrated with gradient ethanol, cleared with xylene, embedded in paraffin, and sliced continuously at a thickness of 3 m. The distribution and arrangement of collagen were observed under a microscope after HE staining. (2) Picrosirius-polarization staining: routine section, dewaxing, and dehydration of specimens were performed. Then, the cells were soaked in Sirius red picric acid staining solution for 1 h, dyed again, cleared, and sealed after washing. A section was taken from each specimen, and the distribution and shape of type I and type III collagen fibers were observed under a polarization microscope. Five fields of vision were randomly selected from each slice at 400 times magnification and photographed at the same exposure. The relative total area of type I collagen fibers (red and yellow) and type III collagen fibers (green) and the ratio of type I/III collagen fibers was obtained by measuring the red, yellow, and green areas with Image Proplus 6.0 image analysis software. The unit of the relative area of collagen fiber was pixels.

#### Statistical analysis

SPSS 21.0 for Windows statistical software was used for statistical analysis. The data are expressed as the mean standard deviation (SD). A *t* test was used to compare the mean of two samples. *P* < 0.05 was considered significant.

## Results

### Comparison of MCL length

At 8 W, 16 W, 24 W, and 40 W after PCL rupture of the knee joint, the MCL of the experimental group and the control group lengthened gradually with time, the length of the MCL in the experimental group was greater than that in the control group at each time point, and the difference was not significant. *P*>0.05 indicated that there was no significant difference in MCL length between the experimental group and the control group at each time point.

### Maximum load and maximum displacement of the MCL

At 8 W, 16 W, and 24 W, the maximum load of the MCL in the experimental group and the control group increased gradually. At 40 W, the MCL load of the control group remained unchanged, but the MCL load of the experimental group decreased. At 8 W, 16 W, and 24 W, the maximum load of the MCL in the experimental group was greater than that in the control group, and the difference gradually decreased with the extension of time. There was no significant difference between the experimental group and the control group at each time point (*P*>0.05). The maximum load of the experimental group at 40 W was smaller than that of the control group, and the difference was significant (*P*<0.05). The results of the maximum load of the knee MCL are shown in Table 1.

**Table 1:** Maximum load of MCL after PCL rupture of rabbit knee joint ($\overline{x}\pm s$, unit: *N*)

Time point	Experimental group	Control group	*P*
8W	63.864.53	56.793.22	>0.05
16W	68.304.29	62.244.69	>0.05
24W	69.025.03	66.223.45	>0.05
40W	60.487.00	66.223.45	<0.05

The maximum displacements of the experimental group and the control group at each time point are shown in Table 2. The maximum displacement of the experimental group at 8 W, 16 W, 24 W, and 40 W was greater than that of the control group. The *t* test for pairwise comparison of the experimental group and the control group at each time point was performed, and the results were all *P* > 0.05. Therefore, it was considered that there was no significant difference in the maximum displacement between the experimental group and the control group at each time point.

**Table 2:** Maximum displacement of MCL after PCL rupture of rabbit knee joint ($\overline{x}\pm s$, unit: mm)

Time point	Experimental group	Control group	*P*
8W	3.390.15	3.310.16	>0.05
16W	3.430.18	3.380.16	>0.05
24W	3.560.16	3.340.09	>0.05
40W	3.540.22	3.410.17	>0.05

### Modulus of elasticity of the MCL

The elastic modulus of the MCL of the experimental group and the control group at each time point after PCL rupture of the knee joint are shown in Table 3. The nano-indentation test of the eight groups of samples showed that the modulus of elasticity of the control group was the smallest at 8 W, which was 3.116 0.267 GPa, while that of the experimental group was the largest at 24 w, which was 3.4400.277 GPa. There was no significant difference in the elastic modulus between the experimental groups and the control group at 8 w and 16 w (*P*>0.05), while there was a significant difference in the elastic modulus between the experimental groups and the control group at 24 W and 40 W (*P*<0.05). The elastic modulus of the MCL in the experimental group and the control group increased gradually at 8 W, 16 W, and 24 W and remained basically the same as that in the control group at 40 W, but the elastic modulus of the MCL in the experimental group decreased. The elastic modulus of the MCL in the experimental group was greater than that in the control group at 8 W, 16 W, and 24 W, while the value in the 40 W control group was greater than that in the experimental group.

**Table 3:** Elastic modulus and micro-hardness of MCL after PCL rupture in rabbit knee joints ($\overline{x}\pm s$, unit: GPa)

Time point	Elastic modulus		Micro-hardness	
	Experimental group	Control group	Experimental group	Control group
8W	3.1540.450	3.1160.267	0.1260.018	0.1240.016
16W	3.3050.274	3.2130.296	0.1410.012	0.1300.020
24W	3.4400.277	3.2580.291*	0.1520.012	0.1400.016*
40W	3.1750.318	3.2880.323*	0.3140.025	0.1450.023*

*P<0.05

### Nano-indentation micro-hardness of the MCL

The MCL micro-hardness of the experimental group and the control group at each time point is shown in Table 3. A nano-indentation test was performed on eight groups of samples. The results showed *P* > 0.05 between the two groups at 8 W and 16 W and *P*=0.040 at 24 W. Therefore, it was considered that the MCL micro-hardness of the experimental group at 24 W was significantly greater than that of the control group. *P* <0.05 indicated that the MCL micro-hardness of the experimental group at 40 W was significantly lower than that of the control group. The MCL micro-hardness of the experimental group and the control group increased gradually at 8 W, 16 W, and 24 W and decreased at 40 W, while the micro-hardness of the control group increased with the passage of time at all time points. The MCL micro-hardness of the experimental group was greater than that of the control group at 8 W, 16 W, and 24 W, while the MCL micro-hardness of the 40 W experimental group was lower than that of the control group.

### HE staining

**Fig. 1:**
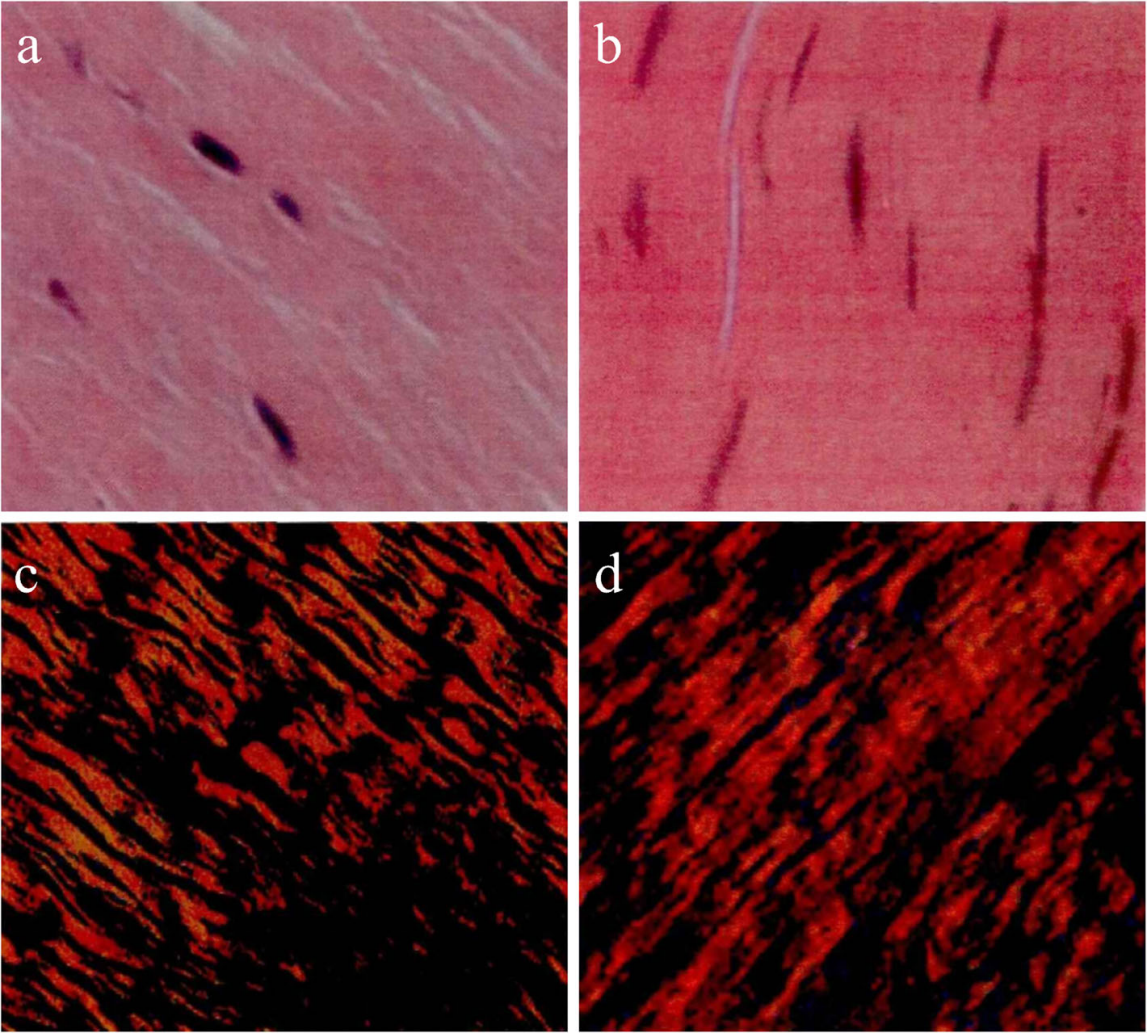
HE staining and picrosirius-polarization staining between the experimental group and the control group at 40 W. **a** In the experimental group, there were some round and oval cells as well as a small number of spindle cells in the MCL at 40 W, presenting with vertical rows, deep color, different sizes, and uneven distribution (HE staining, 400). **b** In the control group, there were some spindle cells in the MCL at 40 W, presenting with vertical rows, deep color, different sizes, and uneven distribution as well as dense extracellular collagen fibers (HE staining, 400). **c** In the experimental group, obviously loose collagen fibers were seen and locally arranged irregularly at 40 W; type III collagen fibers were distributed between type I collagen fiber bundles (picrosirius-polarization staining, 400). **d** In the control group, collagen fibers were arranged regularly and in bundles, and type III collagen fibers were scattered inside the fiber bundles of collagen type I (picrosirius-polarization staining, 400)

There was no significant difference in the MCL between the experimental group and the control group at 8 W, 16 W, and 24 W after PCL rupture. At 40 W, some round and oval and a small number of spindle cells were observed in the experimental group. The cells were arranged in a vertical line. The color of the cells was dark, the size was different, and the distribution was uneven. The collagen fibers outside the cells were small, sparse, and orderly arranged, as shown in Fig. 1a. In the control group, some spindle cells were observed, which were arranged vertically. The color of the cells was dark, the size was different, and the distribution was uneven. The collagen fibers outside the cells were dense and arranged in an orderly manner, as shown in Fig. 1b.

### Picrosirius-polarization staining

Under a polarization microscope, there was no significant difference in the MCL between the experimental group and the control group at 8 W, 16 W, and 24 W after PCL rupture. At 40 W, the collagen fibers in the experimental group were obviously loose, the local arrangement was not neat, and most of them were in the same direction. Type III collagen fibers were distributed between type I collagen fiber bundles, as shown in Fig. 1c. In the control group, the collagen fibers were arranged in an orderly manner, in bundles, and in the same direction. Type III collagen fibers were scattered in the interior of type I collagen fiber bundles, as shown in Fig. 1d.

### Comparison of type I and type III collagen fibers

At 8 W, 16 W, and 24 W, the relative area of type I collagen fibers and type III collagen fibers in the experimental group was larger than that in the control group, and the difference between the experimental group and the control group at 16 W and 24 W was significant (*P* < 0.05). The relative area of type I femoral fibrils in the experimental group was smaller than that in the control group, and the difference was significant (*P* < 0.05). However, the relative area of type III femoral fibrils in the experimental group was smaller than that in the control group, and the difference was not significant (*P* > 0.05). The relative areas of type I collagen fibers and type III collagen fibers at each time point are shown in Table 4.

**Table 4:** Relative area of type I collagen fibers and type III collagen fibers of MCL after PCL rupture in rabbits ($\overline{x}\pm s$, unit: Pixel)

Time point	Type I collagen fiber		Type III collagen fiber	
	Experimental group	Control group	Experimental group	Control group
8W	2288298791121	1965850542089	28752185646	257014120367
16W	2457075441482	2132067623800*	331327107473	25877194562*
24W	2686478573531	2286165748951*	340489119725	281128131748*
40W	1896804638037	2490337634436*	24454889373	28721292497

*P<0.05

At 8 W, 16 W, and 24 W, the relative total area of type I and type III collagen fibers in the MCL of the experimental group was significantly larger than that of the control group (*P* < 0.05) and was significantly smaller than that of the control group at 40 weeks (*P* < 0.05) as shown in Table 5. At each time point, the ratio of type I/III collagen fibers in the MCL of the experimental group was lower than that of the control group, and there was a significant difference between the experimental group and the control group at 16, 24, and 40 weeks (*P* < 0.05) as shown in Table 6.

**Table 5:** Relative total area of type I and type III collagen fibers in MCL after PCL rupture in rabbits ($\overline{x}\pm s$, unit: Pixel)

Time point	Experimental group	Control group	*P*
8W	2575819857442	2222864624240	<0.05
16W	2788403476859	2390838725751	<0.05
24W	3026968674192	2567293846089	<0.05
40W	2141352708207	2777549708652	<0.05

**Table 6:** The ratio of type I/type III collagen fibers in MCL after PCL rupture in rabbits ($\overline{x}\pm s$)

Time point	Experimental group	Control group	*P*
8W	8.122.27	8.522.67	>0.05
16W	8.283.83	8.802.43	<0.05
24W	8.371.86	9.022.83	<0.05
40W	7.981.76	9.071.90	<0.05

## Discussion

There are two main factors affecting the strength of the ligament under load: the shape and size of the ligament and the loading speed [19]. The larger the number of fibers in accordance with the loading direction, and the wider and thicker these fibers are, the stronger the ligament is. In the tensile test, it was found that the maximum load of the MCL of an intact PCL knee joint increased gradually with the extension of time, which increased greatly between 8 W and 16 W, 16 W, and 24 W. Although the time interval was twice as long as before, the increase was relatively slow at 40 W. Woo et al. [20] carried out mechanical tests on the MCL of rabbits and found that the maximum load of rabbits increased with age, and the male rabbits reached the plateau stage at 6 months. At 8 W, 16 W, and 24 W, the maximum MCL load of the PCL rupture knee joint gradually increased, and the maximum MCL load at each time point was greater than that of the intact PCL knee joint. Many studies have shown that mechanical stimulation can change cell performance through a variety of signaling pathways [21]. After removing the MCL of the rabbit, Mark implanted a stainless steel nail under its healing tissue to increase the stress when keeping it in the cage for 4 W [22]. After feeding for a period of time, he found that the total amount of collagen and the ratio of I/III type collagen were closer to normal than those in the natural healing group without the steel nail. Therefore, it is believed that increasing the stress could improve the histological remodeling of the ligament, i.e., the increase in the number of more longitudinally arranged collagen fibers and cells.

Similar to the tensile mechanical results, the nano-indentation test also revealed that the elastic modulus and hardness of the rabbit MCL increased with age. In addition, the elastic modulus and micro-hardness of the PCL-cut knee joint MCL at 8 w, 16 w, and 24 w were greater than those of the PCL-complete group, and there was a significant difference at 24 W. After a rupture of the PCL, the MCL played a compensatory role by increasing the stress, and it was easier to tension or to pull more in the process of the activity. In contrast, under the condition of loaded braking, the mechanical properties will decrease. It was found that the stiffness, ultimate load, and energy absorption of the femoral-MCL-tibia complex of rabbits decreased significantly after braking, and the elastic modulus of the MCL decreased by 50% [23]. The results of Walsh et al. [24] showed that the maturation of the structural characteristics of MCL was inhibited during the immobilization process, and the longer the immobilization time was, the more obvious the sign of structural degradation was. Therefore, it is considered that certain stress stimulation can promote the remodeling of the MCL and increase the mechanical properties to meet the functional requirements. It was also found that the elastic modulus and micro-hardness of the experimental group were significantly decreased compared with those of the control group at 40 W.

The MCL of the rabbit knee joint was strained only during extension and excessive flexion, while the knee joint was flexed when standing still [25]. After the PCL was removed, the knee joint was not fixed, resulting in backward instability. Although rabbits can move freely, the joint load is generally physiological, which is due to the limitation of cage space and knee joint range of motion, so the degree of PCL backward displacement is less than that of severe exercise or overload. In contrast, it stimulates signal changes in cells and extracellular matrix (ECM) in the ligament, which leads to structural remodeling and functional adaptation of the ligament. With the extension of the PCL rupture time, it was found that the maximum load and micro-hardness of the experimental group were significantly reduced compared with those of the control group at 40 W. Although PCL rupture does not cause damage in a short time, the relaxation of soft tissues increases with the extension of time, and the stress on the MCL also increases correspondingly, resulting in slow ligament injury.

Type I collagen of the ligament is composed of tightly arranged and thick collagen fibers, which can resist a high load, while type III collagen comprises small fibers with less strength than type I collagen [26]. Therefore, the histological and material mechanical properties of ligaments can be judged to a certain extent by comparing the relative amount of type I collagen and type III collagen and the ratio of type I/III collagen [27]. Amiel et al. [28] studied the MCL of 2-, 12-, and 36-month-old New Zealand white rabbits and found that with increasing rabbit age, the water content in the ligament decreased significantly, the degree of collagen crosslinking increased, and the collagen synthesis rate decreased from the highest value at 2 months to the lowest value at 36 months. The structure of the MCL in the knee joint gradually tended to mature, and the histological properties were also improved. Some studies have also confirmed that MCL cells have a good ability to synthesize collagen and to induce collagen maturation [29]. Early studies found that type III collagen was first produced in the healing process of ligament tissue and then transformed into type I collagen after a period of time. With the transformation of type III collagen to type I collagen, the tendon and ligament obtained stronger mechanical properties [30]. Busch et al. [31] found that after mechanical stimulation of patellar ligament fibroblasts, the release of type I procollagen and fibrin increased significantly after 6 h, while after mechanical stimulation of fibroblasts for 30 min, the release of type III procollagen increased significantly after 12 h. The viscoelasticity of the ligament can highly adapt to knee joint function, and collagen fiber allows stretching within a certain range, but when the external force exceeds its allowable range or overloads for a long time, fibroblasts or collagen will be damaged [32].

The mechanical properties of the MCL in rabbits did not decrease significantly in the past 6 months after knee rupture but improved to some extent, which suggests that the ligament achieves functional compensation through structural remodeling. However, the maximum load, elastic modulus, and micro-hardness tended to decrease after nearly 10 months of PCL rupture. Histological observation of the MCL revealed that the number of MCL collagen fibers in the PCL rupture group was increased compared with the PCL intact group in the short term, but the ratio of type I/type III collagen fibers was different from that in the PCL intact group, so it was considered that structural remodeling did not completely normalize the ligaments. The number of MCL type I and type III collagen fibers decreased significantly at 40 W, which also explained our previous biomechanical results.

The findings of this study have to be seen in light of some limitations. First, the bilateral knee joints of experimental animals were used as self-controls in this study. Although the control group was also treated with a sham operation, PCL disruption in the experimental group may also affect the control group, which needs to be corrected in future studies. Second, due to the lack of PCL fracture research on the MCL, the results of this study cannot be directly compared with previous studies. This aspect is not only an innovation but also a limitation of this research. Third, this study did not analyze the cartilage or perform microscopic identification of the cartilage, which made it impossible to evaluate the development of OA after PCL rupture. It is worth studying the OA process in the future.

## Conclusion

PCL ruptures do not have a large effect on the mechanical and histological properties of the MCL under short-term physiological loading, but the histological and mechanical properties of the MCL show a significant decrease over time. Therefore, if a PCL rupture fails to effectively restore posterior stability for a long time, even with lower physiological load and limited range of motion, MCL damage can occur. This finding should be given attention in clinical practice.

## Data Availability

The datasets used and analyzed during the current study are available from the corresponding author on reasonable request.

## Abbreviations

PCL: Posterior cruciate ligament
MCL: Medial collateral ligament
ACL: Anterior cruciate ligament
OA: Osteoarthritis
FMTC: Femur MCL tibia complex
ECM: Extracellular matrix
